# The importance of primary tumor origin in gastrointestinal malignancies undergoing cytoreductive surgery and hyperthermic intraperitoneal chemotherapy

**DOI:** 10.1186/s12957-020-01938-0

**Published:** 2020-07-23

**Authors:** Natasha Leigh, Daniel Solomon, Eric Pletcher, Daniel M. Labow, Deepa R. Magge, Umut Sarpel, Benjamin J. Golas

**Affiliations:** Division of Surgical Oncology, Icahn School of Medicine at Mount Sinai St. Luke’s West Hospital, 425 West 59th Street, Suite 7B, New York, NY 10019 USA

**Keywords:** Peritoneal carcinomatosis, Hyperthermic intraperitoneal chemotherapy, Cytoreductive surgery, Gastric cancer, Small bowel cancer

## Abstract

**Background:**

Appendiceal and colorectal cancers with peritoneal carcinomatosis (PC) can derive benefit from cytoreductive surgery and hyperthermic intraperitoneal chemotherapy (CRS/HIPEC). However, its role in gastric and small bowel malignancies remains undefined.

**Methods:**

We retrospectively analyzed 251 gastrointestinal adenocarcinomas with PC which underwent CRS/HIPEC at our institution from 2007 to 2017. We compared outcomes of gastric, small bowel, appendiceal, and colorectal cohorts.

**Results:**

Thirty-one gastric, 8 small bowel, 91 appendiceal, and 121 colorectal cohorts were included. More gastric cancers (90%) received neoadjuvant chemotherapy than any other cohort, *p* = 0.002. Although colorectal had the lowest peritoneal cancer index (PCI) (9) and appendiceal the highest (16), all cohorts underwent similar rates of organ resection and complete cytoreduction. Length of stay (*p* = 0.005) and major perioperative morbidity (Clavien III/IV, *p* = 0.011) were significantly higher in gastric and small bowel. Median overall survival (OS, *p* < 0.001) was significantly shorter in gastric (13 months) and small bowel (9 months) than in appendiceal (33 months) and colorectal (42 months) cohorts. On multivariate analysis, complete cytoreduction and PCI score were significant predictors of OS, *p* < 0.05.

**Conclusions:**

Primary tumor origin significantly affects outcomes after CRS/HIPEC for gastrointestinal malignancies. Though there was a survival benefit in appendiceal and colorectal, gastric and small bowel survival was comparable to systemic chemotherapy.

## Background

Malignancies originating from the proximal gastrointestinal (GI) tract are rare, with 28,000 cases of gastric and 10,190 cases of small bowel cancers diagnosed annually [1]. A significant proportion of these patients have metastatic disease at diagnosis, and current National Comprehensive Cancer Network (NCCN) guidelines recommend palliative chemotherapy and supportive care in this setting [2]. Overall survival (OS) in patients with peritoneal carcinomatosis (PC) secondary to gastric and small bowel malignancies receiving best palliative therapy is poor: 12 months [3] and 17 months [4], respectively.

Improved survival has been previously demonstrated in select patients with PC from multiple other primary tumor origins undergoing cytoreductive surgery and hyperthermic intraperitoneal chemotherapy (CRS/HIPEC) compared to palliative chemotherapy alone [5–12]. With regard to GI adenocarcinomas, multiple authors have documented significant survival benefit in colorectal cancers with PC undergoing CRS/HIPEC (22.2 versus 12.6 months) [7, 8, 13], and as such, this procedure has now been incorporated into the treatment algorithm. Data on appendiceal adenocarcinomas is limited, but studies do seem to suggest that there is still a survival advantage for CRS/HIPEC over palliative chemotherapy (50 versus 20 months) [14, 15].

A few retrospective studies with small sample sizes have investigated the utility of CRS/HIPEC in gastric [3, 16–18] and small bowel [19–21] cancers. However, there remains a paucity of data to support its use in these GI malignancies with PC.

The purpose of this study was to analyze our institutional experience with select GI malignancies with PC undergoing CRS/HIPEC at our high-volume center. We sought to determine if there was a difference in perioperative and survival outcomes depending on the primary tumor origin within the GI tract, and to determine if any survival advantages are seen in gastric and small bowel malignancies with PC.

## Methods

This is a retrospective study of 251 consecutive patients who underwent attempted CRS/HIPEC for PC secondary to GI primaries at a single high-volume institution between July 2007 and May 2017. Patients were grouped according to primary tumor location given the different disease biologies: gastric, small bowel, appendiceal, and colorectal. Only patients with adenocarcinoma histology were included. Patients with any radiographic evidence of metastatic disease outside of the peritoneal cavity were excluded. All patients were discussed in a multidisciplinary tumor board prior to surgery. All procedures were performed by four surgical oncologists with similar surgical experience and who were familiar with CRS/HIPEC. This study was approved by the Mount Sinai School of Medicine institutional review board.

### Surgical procedure

CRS/HIPEC was performed in a standard fashion as previously described [22, 23]. The procedure was aborted if the surgeon felt that the likelihood of successful complete cytoreduction was minimal. The peritoneal cancer index (PCI) was calculated prior to CRS. The completeness of cytoreduction (CC) score was recorded at end of CRS. Complete cytoreduction was defined as CC-0 (no macroscopic disease) or CC-1 (residual tumor implants < 2.5 mm in diameter). Incomplete cytoreduction was defined as CC-2 (residual tumor implants 2.5 mm to 2.5 cm in diameter) or CC-3 (residual tumor implants > 5 cm in diameter). HIPEC was performed using intraperitoneal chemotherapy instilled at 42 °C for 90 min using a closed abdomen technique. Perioperative complications were graded according to the Clavien-Dindo classification system [24] and defined as occurring within 30 days of CRS/HIPEC. Major perioperative morbidity was classified as Clavien-Dindo grade III–IV, and perioperative mortality was classified as Clavien-Dindo grade V.

### Outcomes

The primary endpoints were progression-free survival (PFS) and overall survival (OS). OS was defined as the time from the index CRS/HIPEC procedure to death or last follow-up. PFS was defined as the time from the index CRS/HIPEC procedure to disease progression or relapse (diagnosed on imaging or re-operation). Postoperatively, patients were followed with serial contrast-enhanced cross-sectional imaging, although there were no strict institutional protocols in place. In the majority of patients, however, this consisted of a CT scan every 3 to 4 months for up to 5 years. Secondary endpoints included the rate of complete cytoreduction and major perioperative morbidity.

### Statistical analysis

All statistical analyses were performed using SAS® software, version 9.4 (SAS Institute Inc., Cary, NC, USA). Categorical variables were compared using Fisher’s exact tests and are reported as totals with percentages. Continuous variables were compared using ANOVA tests and are reported as median values with interquartile ranges (IQR). Normality of distribution was assessed using Shapiro-Wilk tests. Kaplan-Meier estimates were used to analyze PFS and OS, and survival curves were calculated using the log-rank test. Cox proportional hazards models were used to create multivariate models for factors predictive of PFS and OS and are reported as hazard ratios (HRs) with 95% confidence intervals (CI). A *p* value of < 0.05 was considered to be statistically significant.

## Results

### Patient characteristics

Our study identified 31 gastric, 8 small bowel, 91 appendiceal, and 121 colorectal (119 colon, 2 rectal) malignancies. Clinicopathologic characteristics are reported in Table 1. There was no statistically significant difference between the four cohorts in terms of age, gender, and ASA score or prior abdominal surgery. Significantly more gastric cancers (90%) underwent neoadjuvant chemotherapy compared to the small bowel (63%), appendiceal (57%), and colorectal cancers (73%), *p* = 0.002. The most frequent neoadjuvant chemotherapy regimens used were FOLFIRI/FOLFOX in gastric, FOLFIRI in small bowel, and FOLFOX in appendiceal and colorectal malignancies. Small bowel (22 months) and colorectal (17 months) malignancies had a significantly longer time from diagnosis to CRS/HIPEC than did appendiceal (7 months) or gastric (10 months).

**Table 1:** Clinicopathologic characteristics

Characteristic	Gastric	Small bowel	Appendiceal	Colorectal	*p* value
	(*n* = 31)	(*n* = 8)	(*n* = 91)	(*n* = 121)	
Age at surgery, years	57 (46–61)	58 (48–63)	55 (49–62)	53 (43–61)	0.456
Gender (male/female)	13 (42)/18 (58)	7 (88)/1 (13)	41 (45)/50 (55)	49 (41)/72 (59)	0.082
ASA score					0.741
I/II	0 (0)/4 (13)	0 (0)/0 (0)	4 (4)/17 (19)	5 (4)/18 (15)	
III/IV	23 (74)/2 (13)	8 (100)/0 (0)	66 (73)/4 (4)	87 (73)/9 (8)	
Prior abdominal surgery	29 (94)	8 (100)	86 (95)	109 (90)	0.877
Neoadjuvant chemotherapy	28 (90)	5 (63)	52 (57)	88 (73)	0.002*
Time from diagnosis to surgery, months	10 (5–15)	22 (2–40)	7 (4–24)	17 (7–31)	0.009*

*ASA* American Society of Anesthesiology

*p < 0.05

### Perioperative outcomes

Table 2 reports perioperative outcomes. The majority of patients across all cohorts underwent successful CRS and HIPEC, with a similar number of aborted procedures. Intraoperatively, the colorectal cohort had the lowest median PCI (9), and appendiceal (16) had the highest (*p* = 0.008), although this did not correlate with a difference in the number of organs resected or the ability to obtain complete cytoreduction. More anastomoses were performed in the gastric and small bowel cohorts, with 13% and 25% (versus 5% appendiceal and 4% colorectal) requiring ≥ 3 anastomoses (*p* < 0.001). In the perioperative period, the gastric and small bowel cohorts exhibited a significantly longer LOS (*p* = 0.005) and higher perioperative major morbidity (*p* = 0.011) than the appendiceal and colorectal cohorts.

**Table 2:** Perioperative outcomes

Value	Gastric	Small bowel	Appendiceal	Colorectal	*p* value
	(*n* = 31)	(*n* = 8)	(*n* = 91)	(*n* = 121)	
Type of surgery					0.186
CRS/HIPEC	24 (77)	6 (75)	70 (77)	105 (87)	
CRS alone	7 (23)	2 (25)	14 (15)	16 (13)	
Aborted procedures	7 (23)	2 (25)	22 (24)	16 (13)	0.146
PCI	14 (5–20)	11 (4–19)	16 (9–24)	9 (5–19)	0.008*
Number of organs resected					0.129
0	6 (19)	0 (0)	25 (28)	17 (14)	
1–7	18 (58)	7 (88)	46 (52)	81 (67)	
8–16	7 (23)	1 (13)	17 (19)	22 (18)	
Number of anastomoses					< 0.001*
0	7 (23)	3 (38)	43 (47)	55 (46)	
1–2	20 (64)	3 (38)	38 (42)	60 (50)	
≥ 3	4 (13)	2 (25)	5 (5)	5 (4)	
CC score					0.057
CC-0/1	22 (71)	5 (63)	61 (67)	95 (79)	
CC-2/3	9 (29)	3 (38)	30 (33)	26 (21)	
EBL, cc	250 (100–600)	250 (150–1450)	200 (50–500)	200 (100–350)	0.383
LOS, days	9 (6–13)	14 (8–15)	5 (4–8)	7 (5–9)	0.005*
Perioperative major morbidity	11 (35)	4 (50)	12 (13)	23 (19)	0.011*
Perioperative re-operation	6 (19)	1 (13)	8 (9)	12 (10)	0.336
Perioperative mortality	0 (0)	0 (0)	0 (0)	2 (2)	0.650
Adjuvant chemotherapy	24 (77)	5 (63)	81 (89)	105 (87)	0.093
Follow-up time, months	7 (1–13)	9 (2–27)	22 (9–34)	18 (4–33)	0.009*
Median OS, months	13 (3–31)	9 (2–NR)	33 (21–NR)	42 (15–NR)	< 0.001*
1-year OS	10 (54)	3 (44)	63 (85)	74 (82)	
3-year OS	2 (14)	3 (44)	20 (45)	27 (54)	
5-year OS	0 (0)	3 (44)	11 (39)	9 (43)	
Median PFS, months	9 (3–13)	7 (2–15)	12 (5–21)	12 (4–33)	0.063
1-year PFS	7 (32)	2 (25)	36 (48)	49 (49)	
3-year PFS	1 (5)	1 (13)	9 (22)	13 (24)	
5-year PFS	1 (5)	0 (0)	6 (19)	5 (15)	

*CRS* cytoreductive surgery, *HIPEC* hyperthermic intraperitoneal chemotherapy, *PCI* peritoneal carcinomatosis index, *CC* completeness of cytoreduction, *EBL* estimated blood loss, *OR* operating room, *LOS* length of stay, *OS* overall survival, *PFS* progression-free survival

*p < 0.05

### Survival outcomes

**Fig. 1:**
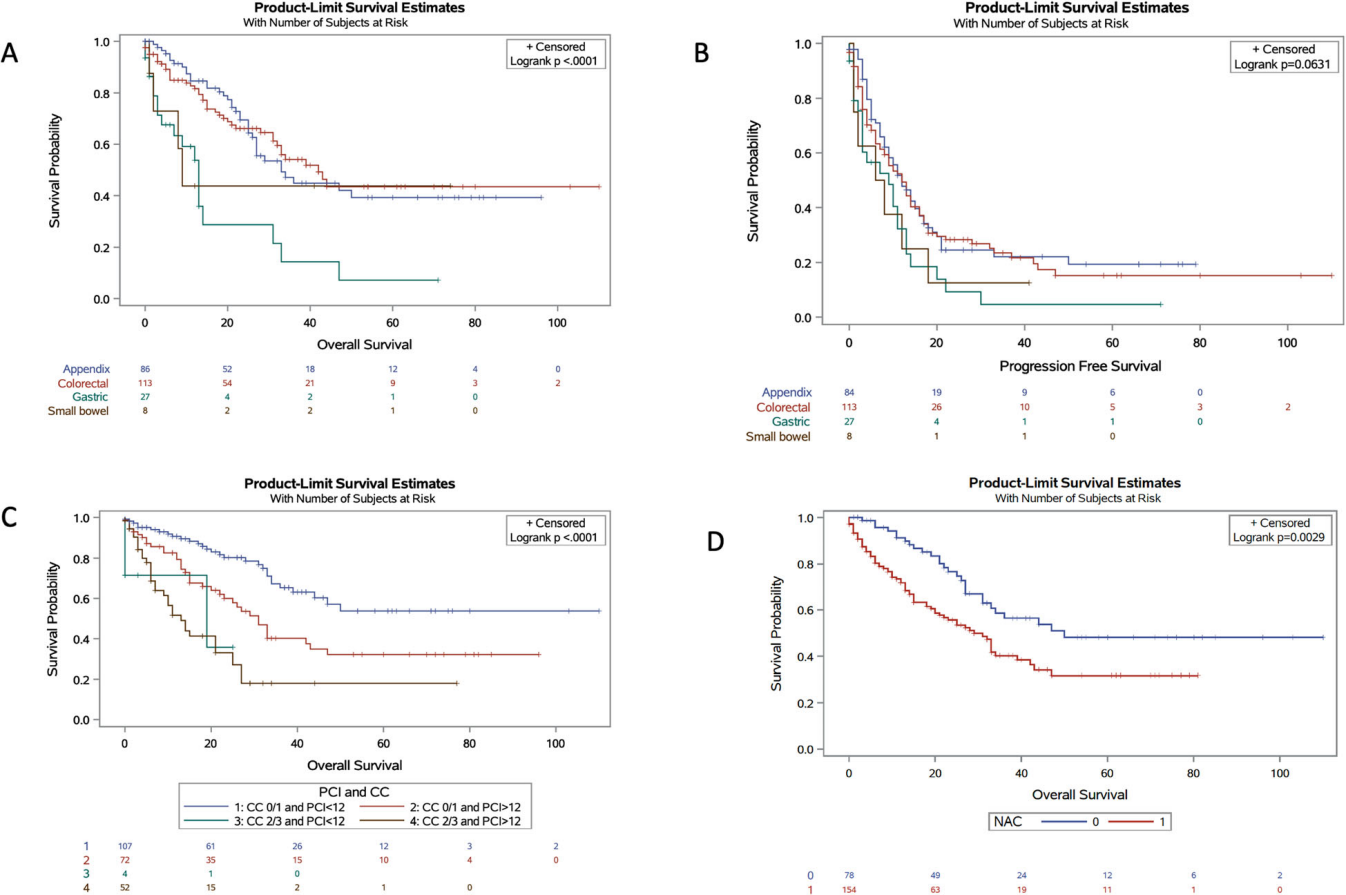
Kaplan-Meier curves for survival. **a** Overall survival (months) for gastric, small bowel, appendiceal, and colorectal cohorts. **b** Progression-free survival (months) for gastric, small bowel, appendiceal, and colorectal cohorts. **c** Overall survival (months) for all patients by CC and PCI scores. **d** Overall survival (months) for all patients by receipt of neoadjuvant chemotherapy

Table 2 reports survival data. The median follow-up time was 15 months, significantly longer in the appendiceal and colorectal cohorts. OS was significantly poorer in patients with gastric (median 13 months; 1-year 54%; 5-year 0%) and small bowel (median 9 months; 1-year 44%; 5-year 44%) primary origins of PC when compared to appendiceal (median 33 months; 1-year 85%; 5-year 39%) or colorectal (median 42 months; 1-year 82%; 5-year 43%), *p* < 0.001 (Fig. 1a). Similarly, gastric and small bowel malignancies had shorter PFS compared to appendiceal and colorectal origins, *p* = 0.063 (Fig. 1b).

Table 3 presents multivariate Cox proportional regression analyses for factors predictive of OS and PFS. Significant predictors of OS were CC-0/1, tumor recurrence, PCI score, PCI < 12, and PFS. Significant predictors of PFS were colorectal PC origin and PCI score.

**Table 3:** Cox proportional hazards models for factors predictive of survival outcomes

Outcome	Variable	Multivariate analysis	
		HR (CI)	*p* value
OS^a^	PC primary tumor origin		
	Gastric	1.60 (0.52–4.88)	0.411
	Appendiceal	0.34 (0.11–1.02)	0.054
	Colorectal	0.45 (0.15–1.33)	0.150
	Complete cytoreduction	0.27 (0.12–0.63)	< 0.001*
	Tumor recurrence	2.73 (1.58–4.73)	< 0.001*
	PCI score	1.08 (1.03–1.14)	0.002*
	PCI < 12	2.28 (1.01–5.12)	0.047*
	PFS	0.89 (0.87–0.92)	< 0.001*
PFS^b^	PC primary tumor origin		
	Gastric	0.84 (0.36–1.98)	0.694
	Appendix	0.45 (0.21–1.00)	0.230
	Colorectal	0.62 (0.29–1.35)	0.049*
	PCI score	1.05 (1.03–1.07)	< 0.001*

*HR* hazard ratio, *CI* confidence intervals, *OS* overall survival, *PC* peritoneal carcinomatosis, *PCI* peritoneal cancer index, *PFS* progression-free survival

*p < 0.05

Univariate non-significant predictors excluded from multivariate analysis

^a^Neoadjuvant chemotherapy, length of stay, adjuvant chemotherapy, perioperative complication

^b^Neoadjuvant chemotherapy, PCI < 12, complete cytoreduction, length of stay, adjuvant chemotherapy, perioperative complication

When all patients were stratified by completeness of cytoreduction and PCI score, those with both CC-0/1 and PCI < 12 had a significantly longer median OS (not reached > 110 months, Fig. 1c) than either CC-0/1 alone (31 months) or PCI < 12 alone (19 months), *p* < 0.001. When patients were then substratified by PC primary tumor origin, the same results were seen; OS was significantly longer for PCI < 12 and CC-0/1 cohorts, for all primary PC origins (gastric 31 months, small bowel not reached > 41 months, appendiceal 50 months, colorectal not reached > 110 months).

When all patients were stratified by receipt of neoadjuvant chemotherapy, those who did not receive chemotherapy had a significantly longer median OS (50 months) than those who did receive chemotherapy (29 months), *p* = 0.003 (Fig. 1d). Again, when substratified by PC primary tumor origin, OS was significantly longer for patients who did not receive neoadjuvant chemotherapy, regardless of primary PC origin (gastric 31 versus 13 months, small bowel not reached > 74 versus 8 months, appendiceal 50 versus 27 months, colorectal not reached > 110 versus 39 months).

## Discussion

### Significance of primary tumor origin

CRS/HIPEC has documented survival advantages for colorectal primaries with PC compared with systemic chemotherapy [6, 8, 13]. A randomized controlled trial by Verwaal et al. demonstrated a significant improvement in PFS (12.6 versus 7.7 months) and median survival (22.2 versus 12.6 months) in patients undergoing CRS/HIPEC using mitomycin C [7]. The data is more limited for appendiceal adenocarcinomas as most of the studies are heterogeneous and include other tumor types (low-grade appendiceal mucinous neoplasms (LAMN), carcinoid). In 2012, Lieu et al. [15] reported on 142 patients with appendiceal adenocarcinomas and found that patients who underwent systemic chemotherapy had significantly poorer OS than those who underwent CRS/HIPEC (1.7 versus 4.2 years, *p* < 0.001). These findings were echoed in 2018 by Aziz et al. [14] who reported on 65 appendiceal adenocarcinomas which underwent CRS/HIPEC and found a 5-year OS of 55%. In our study, we found similar results with a median OS of 33 months and 42 months for all comers with appendiceal and colorectal primaries, respectively. Currently, CRS/HIPEC is an accepted treatment for PC from colorectal or appendiceal cancers (primarily LAMNs), with clear benefits over palliative therapy alone.

Despite advances in systemic therapies and surgical techniques, PC secondary to gastric and small bowel malignancies still confers poor outcomes and remains a frustrating treatment dilemma. Traditional palliative therapies offer meager survival benefits [25, 26], and there exists a need for innovative therapies. The success of CRS/HIPEC in select patients with appendiceal and colorectal primaries has led to increased interest in investigating its potential benefits for other GI primaries. In recent years, there have been a few small retrospective studies examining the outcomes of CRS/HIPEC in gastric and small bowel malignancies with PC. However, to date, its utility in these settings is not well known.

The prognosis of metastatic gastric cancer is dismal, with an OS of 8–12 months with palliative chemotherapy [3]. Though many studies have reported on the outcomes after CRS/HIPEC for PC secondary to gastric cancer, currently, there are insufficient data to recommend its routine use [3, 27]. A 2011 randomized controlled trial by Yang et al. concluded that CRS/HIPEC offered a 70% improvement in median survival [17]. This was echoed in a systematic review by Gill et al. who reported a 5-year OS of 13% with a median OS of up to 15 months [18]. In our study, for all comers with gastric cancer primaries, we found the median OS to be 13 months, comparable to systemic chemotherapy.

Metastatic small bowel adenocarcinoma treated with traditional systemic chemotherapy has a poor prognosis with a median OS and PFS of 13 months and 4.6 months, respectively [4]. In 2002, Marchettini and Sugarbaker [28] were the first to report a small series of six patients who underwent CRS/HIPEC for metastatic small bowel adenocarcinoma and found an OS of 12 months. Since then, other series have demonstrated a wide range of OS times from 12 to 36 months [19–21]; the longer times are comparable to those undergoing CRS/HIPEC for PC from colorectal and appendiceal cancers. Liu et al. found that small bowel non-adenocarcinoma may also benefit from CRS/HIPEC [19]. In our study, for all comers with small bowel primaries, we report a median OS of 9 months, no better than systemic chemotherapy.

Clearly, the primary tumor origin within the GI tract appears to have a huge influence on how these malignancies behave. It is likely that the difference in tumor biology is the major contributing factor to the OS differences in our study, with small bowel tumors being the most aggressive in nature with poorest prognosis. Although it did not reach significance on multivariate analysis, on univariate analysis, primary tumor origin was a significant predictor of OS in our patient cohort.

### Significance of tumor burden

In patients undergoing CRS/HIPEC for PC, the most important factors determining survival are tumor burden and completeness of cytoreduction, irrespective of tumor origin. Liu et al. [19] found that CC-0/1, PCI < 15, and HIPEC were predictive of a better prognosis in small bowel cancer, findings similar to a prior study conducted by Bilimoria et al. [25]. A 2015 meta-analysis by Coccolini et al. [29] resonated these conclusions in gastric cancer, finding that though median survival was similar compared to systemic therapies, when patients were stratified by completeness of cytoreduction, CC-0/1 was a significant predictor of improved survival. Similar results from Fugazzola et al. [16] concurred, finding that a PCI < 12 and complete cytoreduction had a significant impact on prognosis. We also found similar results with PCI < 12 and CC-0/1 being predictive of improved OS. Patients in all cohorts with both CC-0/1 and PCI < 12 had significantly longer survival, including gastric and small bowel malignancies. Therefore, it could be reasonable to consider the application of CRS/HIPEC for patients with gastric or small bowel primaries provided they have a PCI < 12 and are able to achieve complete cytoreduction. Unfortunately, however, as evidenced by our study, over half of the gastric patients had a PCI > 14 and over half of the small bowel patients had a PCI > 11 at the time of surgery, so the application of CRS/HIPEC in these patients will likely be limited. In the colorectal and appendiceal cohorts, there was still a survival advantage over systemic chemotherapy even in the cohorts who had a PCI > 12 or CC-2/3 (but not both); hence, it seems reasonable to attempt CRS/HIPEC in these tumors even if they have a high tumor burden or complete cytoreduction cannot be achieved. The same was not true for gastric or small bowel primaries.

### Significance of HIPEC agent

The most efficacious HIPEC agent in colorectal and appendiceal malignancies is mitomycin C, followed by cisplatin. These results have been extrapolated, and currently these two chemotherapeutic drugs are the most frequently used HIPEC agents in gastric [3, 17, 18, 29–31] and small bowel malignancies [19–21, 28] with PC either as a single agent or in combination [32]. There are no studies comparing the morbidity and efficacy of either drug or their combination, though meta-analyses do not appear to demonstrate any notable survival differences. Thus, it is unlikely that the HIPEC agent itself is a major contributing factor to the wide range of OS reported in the literature for gastric and small bowel malignancies. In this study, mitomycin C was the sole HIPEC agent used, and OS was comparable to prior reported literature using mitomycin C, cisplatin, or both agents.

### Significance of neoadjuvant chemotherapy

Recent studies have proposed the utility of neoadjuvant chemotherapy prior to CRS/HIPEC in an attempt to reduce the tumor burden prior to surgery to increase the likelihood of achieving complete cytoreduction. Multiple studies on ovarian cancer have shown mixed results, and even those which demonstrated an improvement in CC-0/1 rate after neoadjuvant chemotherapy failed to show a difference in OS [33–35]. However, there is a lack of data examining for GI malignancies. Our study demonstrated that patients who underwent neoadjuvant chemotherapy had a significantly poorer OS than those who underwent upfront CRS/HIPEC, irrespective of primary tumor origin. It is possible that upfront CRS/HIPEC, especially in patients with a low PCI where CC-0/1 will likely be obtained, could provide a survival advantage over neoadjuvant chemotherapy. A randomized controlled trial comparing neoadjuvant chemotherapy to upfront CRS/HIPEC with large patient populations is needed to further evaluate this.

Our data suggest that primary tumor origin within the GI tract has a significant influence on outcomes for patients undergoing CRS/HIPEC. We found a survival benefit for patients with PC secondary to appendiceal or colorectal cancer undergoing CRS/HIPEC when compared to systemic chemotherapy. However, our data does not support the routine use of CRS/HIPEC in gastric and small bowel malignancies; the survival outcomes are not superior to current palliative treatments, and the associated perioperative morbidity and mortality are significant. There may be a benefit in select patients with gastric or small bowel malignancies who have a very limited peritoneal disease burden (PCI < 12) and are able to undergo complete cytoreduction.

However, any survival benefits must be weighed against the potential morbidity and mortality from CRS/HIPEC, especially in patients with already such limited life expectancy. Major perioperative morbidity has been reported as high as 100% [16], especially with more extensive resections. Our major morbidity rates in appendiceal and colorectal malignancies were relatively low. Our institution is a high-volume tertiary referral center for patients with PC, which may explain the low complication rates [22]. We report a significantly higher perioperative major morbidity rate in the gastric (35%) and small bowel (50%) cohorts. Given that there was no difference in patient comorbidities, organ resection, CC score, or EBL, it is likely that this can be attributed to the significantly higher number of anastomoses performed in these two cohorts, which likely led to a higher perioperative leak rate, reoperation, and longer LOS. Unfortunately, the data for perioperative leaks was not available for analysis.

The small sample size of the gastric and small bowel cohorts, inherent to the diseases themselves, is a limitation of this study. The follow-up time was also shorter, particularly in these two cohorts. Our study is also limited by its retrospective nature. Further larger prospective studies with longitudinal evaluation need to be conducted in order to determine if there is a role for CRS/HIPEC in gastric and small bowel malignancies with PC. The most likely subset of patients to benefit would be those with either positive peritoneal washings without gross disease or limited peritoneal disease burden with extremely low PCI scores in whom complete cytoreduction can be achieved. The optimal HIPEC agent also needs to be defined.

## Conclusions

Select patients with PC secondary to colorectal and appendiceal malignancies exhibit prolonged survival with CRS/HIPEC. This survival benefit is not seen when CRS/HIPEC is routinely employed in patients with PC secondary to gastric or small bowel malignancies. Highly select patients with PC from gastric and small bowel primaries who have a low peritoneal tumor burden and undergo complete cytoreduction may benefit from CRS/HIPEC; however, additional larger studies are required to fully delineate its utility in this capacity.

## Data Availability

The datasets generated during and/or analyzed during the current study are available from the corresponding author on reasonable request.

## Abbreviations

CC: Completeness of cytoreduction
CI: Confidence intervals
CRS: Cytoreductive surgery
EBL: Estimated blood loss
GI: Gastrointestinal
HR: Hazard ratio
HIPEC: Hyperthermic intraperitoneal chemotherapy
IQR: Interquartile ranges
LOS: Length of stay
LGI: Lower gastrointestinal
NCCN: National Comprehensive Cancer Network
NAC: Neoadjuvant chemotherapy
OS: Overall survival
PCI: Peritoneal cancer index
PC: Peritoneal carcinomatosis
PFS: Progression-free survival
UGI: Upper gastrointestinal
